# Human hematopoietic microenvironments

**DOI:** 10.1371/journal.pone.0250081

**Published:** 2021-04-20

**Authors:** Helene Bjoerg Kristensen, Thomas Levin Andersen, Andrea Patriarca, Klaus Kallenbach, Birgit MacDonald, Tanja Sikjaer, Charlotte Ejersted, Jean-Marie Delaisse

**Affiliations:** 1 Department of Clinical Cell Biology, Institute of Regional Health Science, University of Southern Denmark, Lillebaelt Hospital, Vejle, Denmark; 2 Department of Pathology, Clinical Cell Biology, Odense University Hospital, Odense, Denmark; 3 Department of Clinical Research and Department of Molecular Medicine, University of Southern Denmark, Odense, Denmark; 4 Department of Forensic Medicine, Aarhus University, Aarhus, Denmark; 5 Division of Hematology, Department of Oncology, Hospital "Maggiore della Carità", Novara, Italy; 6 Department of Pathology, Zealand University Hospital, Roskilde, Denmark; 7 Department of Endocrinology and Internal Medicine, Aarhus University Hospital, Aarhus, Denmark; 8 Department of Endocrinology, Odense University Hospital, Odense, Denmark; Universita degli Studi di Parma, ITALY

## Abstract

Dormancy of hematopoietic stem cells and formation of progenitors are directed by signals that come from the bone marrow microenvironment. Considerable knowledge has been gained on the murine hematopoietic stem cell microenvironment, while less so on the murine progenitor microenvironment and even less so on these microenvironments in humans. Characterization of these microenvironments is decisive for understanding hematopoiesis and finding new treatment modalities against bone marrow malignancies in the clinic. However, it is equally challenging, because hematopoietic stem cells are difficult to detect in the complex bone marrow landscape. In the present study we are characterizing the human hematopoietic stem cell and progenitor microenvironment. We obtained three adjacent bone marrow sections from ten healthy volunteers. One was used to identify a population of CD34^+^/CD38^-^ “hematopoietic stem cells and multipotent progenitors” and a population of CD34^+^/CD38^+^ “progenitors” based on immunofluorescence pattern/intensity and cellular morphology. The other two were immunostained respectively for CD34/CD56 and for CD34/SMA. Using the combined information we performed a non-computer-assisted quantification of nine bone marrow components (adipocytes, megakaryocytes, bone surfaces, four different vessel types (arteries, capillaries, sinusoids and collecting sinuses), other “hematopoietic stem cells and multipotent progenitors” and other “progenitors”) within 30 μm of “hematopoietic stem cells and multipotent progenitors”, “progenitors”, and “random cell profiles”. We show that the microenvironment of the “hematopoietic stem cells and multipotent progenitors” is significantly enriched in sinusoids and megakaryocytes, while the microenvironment of the “progenitors” is significantly enriched in capillaries, other “progenitors”, bone surfaces and arteries.

## Introduction

In 1978, Schofield [1] hypothesized that hematopoietic stem cells (HSCs) are regulated by cells in the microenvironment. Since then the importance of this microenvironment, or niche, has been demonstrated in a number of studies [2–17]. Within the last decade, murine studies have shown that the development and treatment of myeloid bone marrow (BM) malignancies originating from the hematopoietic stem and progenitor cells (HSPCs), such as myelodysplastic syndromes, acute myeloid leukemia, and myeloproliferative neoplasms, are not only dependent on factors intrinsic to the malignant cells, but also on the BM microenvironment [18–20].

Our ability to develop new treatment modalities targeted against the malignant microenvironment in humans, implies specific knowledge of the human microenvironment. However, exactly this knowledge is sparse, since most human stem cell studies are performed on aspirated cells using flow cytometry. Moreover, the available studies performed on BM biopsies point in different directions, with one study indicating that the majority of HSPCs are positioned close to sinusoids and mesenchymal stromal cells (MSCs) [8], while other studies indicate that HSPCs are mainly positioned close to bone surfaces [3,9,16]. On the other hand, there is a considerable volume of knowledge in mice. This knowledge strongly supports that the major HSC regulatory BM components are the MSCs and the sinusoids [2,6,7,14,15], whereas a subset of the progenitors are positioned close to bone surfaces [7]. Closely associated to the sinusoids are the megakaryocytes that have been shown to be a part of the HSC niche in mice [4,17], while we are still lacking specific knowledge about this in humans.

In animals, sinusoids occupy 3–6 times as much BM area as the arterial part of the circulation, and they are unique for the hematopoietic BM [21]. The importance of sinusoids for hematopoietic BM function is substantiated by observations in humans, showing that the microvascular bed of the hematopoietic BM consists mainly of sinusoids, while that of hematopoietic-inactive BM adipose tissue consists mainly of capillaries [22]. Moreover, because increased oxygen tension leads to HSC differentiation [23], it is important to acknowledge that sinusoids represent the venous part of the BM circulation. Furthermore, direct measurement of local oxygen concentration in live animals has shown that the perisinusoidal part of the BM is the most hypoxic, while the highest oxygen tension is found in the endosteal part rich in arterial vessels [24]. These findings fit our previous 3D reconstructions and 2D quantifications of a rich capillary network close to human bone surfaces [25–27].

Hematopathologists use several methods to identify normal and malignant human HSPCs. One of the methods is HSPC identification on immunohistochemically-stained adjacent sections from decalcified BM biopsies, using a number of markers, including CD34 and CD117 [28]. While CD34 is a crucial marker [29], the usability of CD117 regarding identification of non-malignant HSPCs can be questioned, because CD117 also is expressed on a range of more differentiated cells, including NK cells, T-cells, erythroid cells and mast cells [30]. An alternative marker combination deserving attention is CD34 and CD38 [31]. Already in the 1990’s several papers demonstrated that aspirated CD34^+^ HSPCs could be divided into an early CD34^+^/CD38^-^ HSC and multipotent progenitor (MPP) population, hereafter mentioned as HSCs/MPPs, and a more differentiated CD34^+^/CD38^+^ population of progenitors [32–34]. The continued relevance of the CD34/CD38 marker combination is exemplified in two recently published studies regarding the prognostic significance of a high CD34^+^/CD38^-^ stem cell burden in patients with myelodysplastic syndromes and acute myeloid leukemia [35,36]. Despite of this promising potential, the CD34/CD38 combination has never been included in the daily practice regarding identification of HSPCs on sections; maybe because immunohistochemistry is poorly suited for identification of different markers in the same cell.

In the present study, we used CD34/CD38 immunofluorescence staining intensity and positioning, combined with a detailed morphological assessment to identify respectively the HSCs/MPPs and the progenitors in the normal human BM. Once these cells were identified, we characterized their microenvironment on immunofluorescence- and immunohistochemically-stained adjacent sections. Hereby, we show that the microenvironment of the HSCs/MPPs is significantly enriched in sinusoids and megakaryocytes, while the microenvironment of the progenitors is significantly enriched in capillaries, other progenitors, bone surfaces and arteries.

## Materials and methods

### Human BM biopsies

We included ten paraffin-embedded decalcified iliac crest BM biopsies from healthy female volunteers. The women were recruited specifically for the present study on the criterion that they were healthy (full medical review, normal blood count, normal DEXA-scan, and without any history of either oncological or hematological disease). They had an age of respectively 36, 41, 41, 50, 57, 58, 58, 59, 67, and 71 years, mean age of 54 years. The study was approved by the Danish National Committee on Biomedical Research Ethics project ID # S-20110112. Fixation, decalcification, and dehydration were performed as previously described [26].

### Immunohistochemistry and immunofluorescence

From each of the ten biopsies three 3.5 μm thick adjacent sections were obtained. The sections were stained respectively: i) with CD34 and CD56, using immunohistochemistry, ii) with CD34, CD38, and tartrate resistant acid phosphatase (TRAcP), using immunofluorescence, and iii) with CD34 and smooth muscle actin (SMA), using immunohistochemistry. This setup was designed to identify CD34^+^ cell profiles, CD34^+^, and CD38^+^ cell profiles with staining overlap, as well as BM components, including bone surfaces with TRAcP^+^ osteoclasts, and CD56^+^ and SMA^+^ osteoblast lineage cell profiles, vasculature with CD34^+^ endothelial cell profiles, and when present SMA^+^ smooth muscle cell (SMC) profiles. The outline of the BM components, including arteries, capillaries, sinusoids, collecting sinuses, megakaryocytes, adipocytes and bone surfaces, could be followed on the immunofluorescence-brightfield images and the adjacent immunohistochemically-stained sections. This combined approach was chosen because immunohistochemistry surpasses brightfield imaging in regards to visualization of the microenvironment.

The sections intended for immunohistochemistry- and immunofluorescence-staining went through stepwise deparaffinization, blocking of endogenous peroxidase activity, rehydration, epitope retrieval, and blocking of unspecific adhesion [26]. i) in order to double-stain for CD34 and CD56 the sections were incubated with a cocktail of two primary antibodies: Monoclonal mouse IgG1 antibody against CD34 class II (clone QBend 10; Agilent, Santa Clara, CA US), and monoclonal rabbit antibody against CD56 (clone EPR2566; Abcam, Cambridge, UK). The CD34-binding mouse antibody was detected with alkaline-phosphatase (AP) polymer conjugated to goat anti-mouse (BrightVision; Immunologic, Duiven, The Netherlands) and visualized with liquid permanent red (LPR; Agilent), and the CD56-binding rabbit antibody was detected with horseradish peroxidase (HRP) polymer conjugated to goat anti-rabbit (BrightVision; Immunologic), and visualized with 3.3’-Diaminobenzidine (DAB; Agilent). ii) in order to double-stain for CD34 and SMA, the sections were first incubated with the mentioned IgG1 antibody against CD34 class II (clone QBend 10; Agilent), which was detected and visualized in the same way as described. After visualization and detection, the IgG1 antibody against CD34 class II and the AP polymer conjugated to goat anti-mouse were removed from the sections by keeping them in buffer at 60°C overnight. This was performed to avoid unspecific binding with the next primary and secondary antibody, while at the same time maintaining the staining. Then they were incubated with a monoclonal mouse IgG2a antibody against SMA (Clone 1A4; Agilent), which was detected with HRP polymer conjugated to goat anti-mouse (BrightVision; Immunologic) and visualized with DAB (Agilent). Finally the double- immunohistochemistry-stained sections were counterstained with Mayer’s hematoxylin and mounted. iii) in order to triple stain, using immunofluorescence detection, the sections were incubated with a cocktail of three different antibodies: The mentioned IgG1 antibody against CD34 class II (clone QBend 10; Agilent), monoclonal rabbit antibody against CD38 (clone EPR4106; Abcam), and monoclonal mouse IgG2b antibody against TRACP (clone 9C5; Merck, Darmstadt, Germany). The CD34-binding antibody was detected with Cy-3 goat anti-mouse IgG1 (Jackson, Suffolk, UK), the CD38-binding antibody was detected with Alexa Flour (AF)-568 goat anti-rabbit IgG (ThermoFisher Scientific, Waltham, MA US), and the TRAcP-binding antibody was detected with AF-488 goat anti-mouse IgG2b (ThermoFisher Scientific). The detection was performed with the sections covered in tinfoil to avoid fading. Finally, the sections were counterstained with Hoechst and mounted.

The high quality of the staining can be appreciated in the figures. Negative controls were systematically performed by omitting the primary antibody. Positive controls for CD34, CD56, and SMA were performed as previously described [26], and for CD38 it was performed on malignant plasma cell profiles. To keep the immunofluorescence staining procedure as short as possible, and thereby minimizing morphological changes, we chose not to include a lineage marker cocktail.

### Image analysis

After counterstaining and mounting, the sections were immediately scanned (NanoZoomer XR; Hamamatsu, Hamamatsu City, Shizuoka Pref., 430–8587, Japan). The image analysis was performed using the NDP.view2 software (Hamamatsu). The figures were assembled using CorelDRAW Graphics Suite X4 (CorelDRAW, Fremont, CA, US) and Microsoft PowerPoint 2010 (Microsoft, Redmond, WA, US).

### HSPC identification and HSPC microenvironment quantification

**Fig 1:**
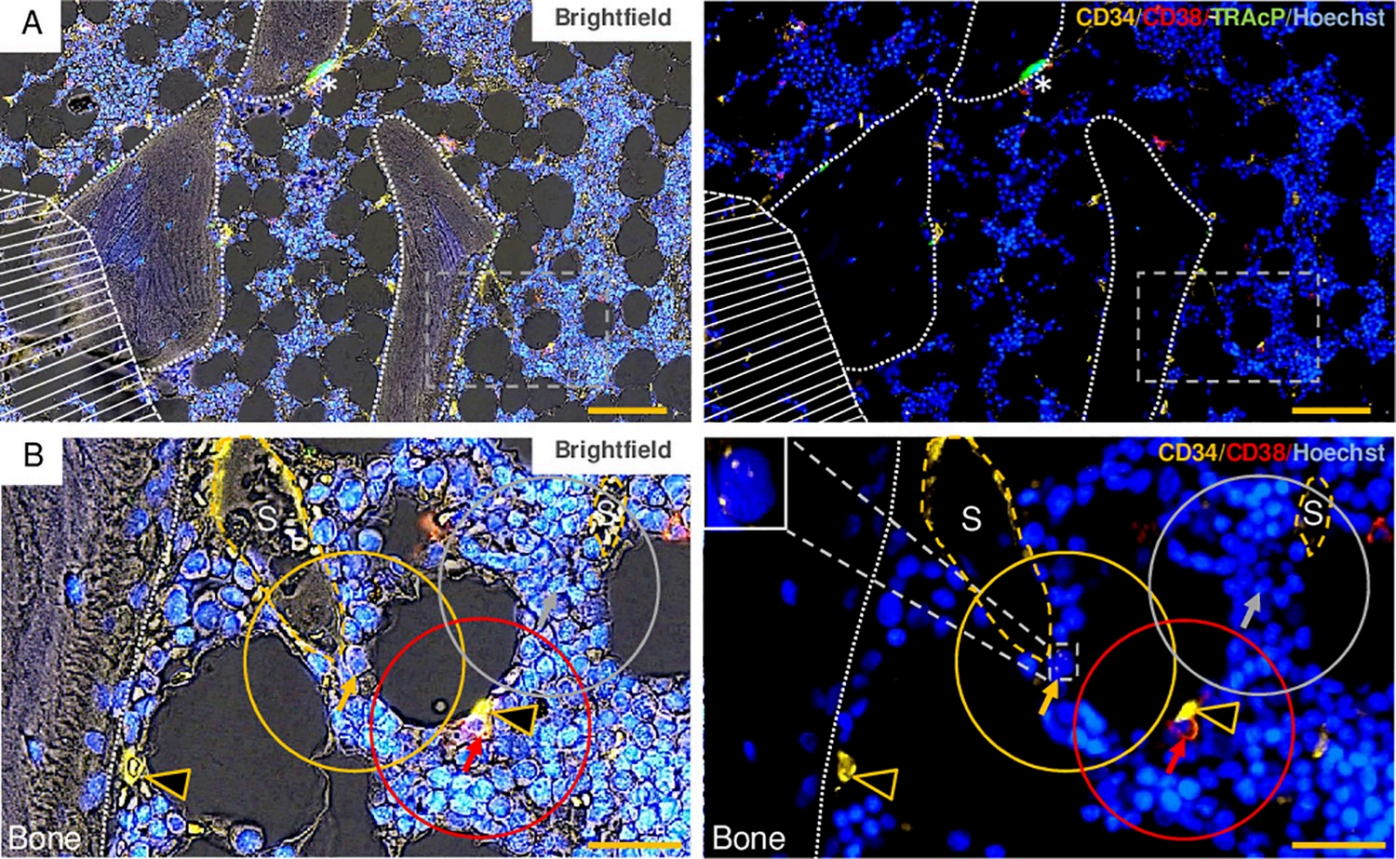
Illustration of the method used to quantify the BM components within a distance of 30 μm from the HSPCs and the random cell profiles. (A) The same field of the section is shown in brightfield (left) and pure fluorescence (right). Note that the representative brightfield image allows identification of the bone matrix and cellular membranes. The hatched area indicates a part of the field which was excluded due to poor quality. White dotted lines indicate outlines of bone surfaces, and the asterisks indicate a TRAcP^+^ osteoclast. (B) Enlargement of the dashed-lined boxed areas in panel A. The same part of the field is shown in brightfield (left) and pure fluorescence (right). The yellow, red, and grey arrows indicate respectively a CD34^+^/CD38^-^ HSC/MPP, a CD34^+^/CD38^+^ progenitor, and a random cell profile. The yellow, red, and grey circles indicate the distance of 30 μm from the outer perimeter of the respective cell profiles. The dashed-line boxed area (right) indicates the HSC/MPP, which is shown at higher magnification in the upper left corner. Capillaries (yellow arrowheads), sinusoids (S and dashed yellow lines). Scale bars = 100 μm in panel A, and = 30 μm in panel B.

In the ten immunofluorescence-stained sections approximately half of the section belonging to the central longitudinal part was selected for further analysis. Parts of the sections in poor quality were outlined and excluded (Fig 1A). A systematic random grid was used to identify random cell profiles throughout the preselected central part of the section. A random cell profile was selected when the grid intersected any cell profile with a distinct nucleus apart from megakaryocytes, HSPCs, adipocytes, bone matrix, endosteal cell profiles, blood vessels and cell profiles positioned in the lumen of blood vessels. An approximately equal number of random cell profiles and HSPCs were included from each section. We identified the HSPCs according to a set of criteria (see results). These criteria were established as a consensus between HBK (senior registrar in hematology, post.doc.), BMD (pathology-trained biomedical laboratory scientist), and KK (hematopathologist, Ph.D.), and were then used when HBK together with AP (hematologist) evaluated the whole HSPC population. Each HSPC was assigned with an individual ID-number to identify them during the following quantification. The quantification of the nine different BM components within 30 μm of all HSPCs and random cell profiles was performed non-computer-assisted using, a circular grid (Fig 1B). The nine different BM components included adipocytes, megakaryocytes, bone surfaces, four different vessel subtypes (arteries, capillaries, sinusoids and collecting sinusoids), and other HSPCs subdivided into HSCs/MPPs and progenitors. To be noted, we defined an artery as any vessel with at least one layer of SMCs. The distance of 30 μm was chosen because previous papers have highlighted the importance of the distance of 25–40 μm concerning the BM oxygen gradient and the interactions between the HSCs and the microenvironment [4,17,24].

### Statistics

Statistics were calculated in STATA using the clustered logistic regression. The clustered logistic regression includes all individual observations from all ten BM biopsies, while at the same time taking into account that the observations belong to separate clusters (each patient represents a cluster). The odds ratio (OR), the level of significance, and the confidence intervals are indicated when there was a significant likelihood to observe a given BM component within a distance of 30 μm from either HSCs/MPPs or progenitors, compared to random cell profiles, or HSCs/MPPs compared to progenitors. When the likelihood was increased the OR is above 1, and when the likelihood was decreased the OR is below 1. Because of multiple sampling the level of significance was adjusted according to a conservative Bonferroni correction, resulting in corrected P-values where * P ≤ 0.01, ** P ≤ 0.002, *** P ≤ 0.0002. Furthermore, to allow a visual overview of the presence of the BM components next to the three investigated cell profile populations, the percentage of the cells within a distance of 30 μm from each of the BM components are depicted as lines.

## Results

### Classification of HSPCs

**Fig 2:**
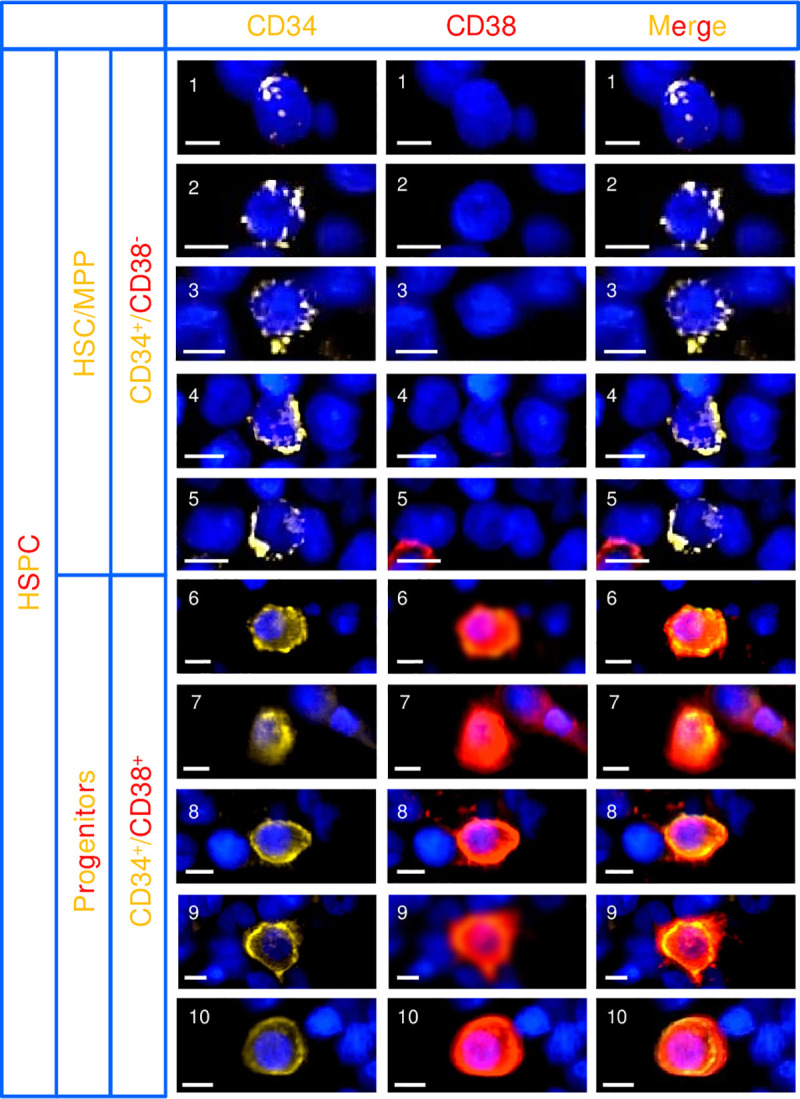
HPSC classification illustrated through images of ten different CD34 and CD38 multiplex-stained cell profiles. The representative images are obtained from five different biopsies: Images 1 and 6, 2 and 7, 3 and 8, 4 and 9 as well as 5 and 10 are obtained from the same biopsy. Cells 1–5 are HSCs/MPPs, while cells 6–10 are progenitors. Note that CD34 distributes in dense fluorescent pockets in cell profiles 1–5, as a partial halo in 2, 3 and 5, and as a perfect halo in 6–10. Respectively, one and two more differentiated cell profiles are present at the periphery of image 5 and 7. Scale bars = 5 μm.

HSPCs were classified according to cellular morphology, as well as to CD34- and CD38-immunofluorescence intensity and pattern (Fig 2). A cell profile was classified as an HSC/MPP when it fulfilled the following criteria: i) a representative nuclear profile; ii) intermediately to intensively positive for CD34 and negative for CD38; iii) high nucleus/cytoplasm ratio; iv) sparse CD34 fluorescence that distributes in dense fluorescent pockets and, depending on how the cell profile was sectioned, as a perinuclear partial halo (Fig 2) [37,38]. A cell profile was classified as a progenitor when it fulfilled the following criteria: i) a representative nuclear profile; ii) intermediately positive for CD34 and intermediately to intensively positive for CD38; iii) intermediate nucleus/cytoplasm ratio; iv) CD34 and CD38 fluorescence distributed evenly as an unbroken halo around at least 25% of the nucleus (the 25% criteria was developed to allow inclusion of cell profiles that were not sectioned through the center, but to avoid inclusion of those that were too marginally sectioned) (Fig 2). The total number of identified HSPCs was 1430, of which 513 were HSCs/MPPs, and 917 were progenitors. In addition to this, a third cell population of 1361 random cell profiles was included for comparison.

### The HSCs/MPPs have greater chances than the progenitors to have sinusoids or megakaryocytes in their microenvironment

**Fig 3:**
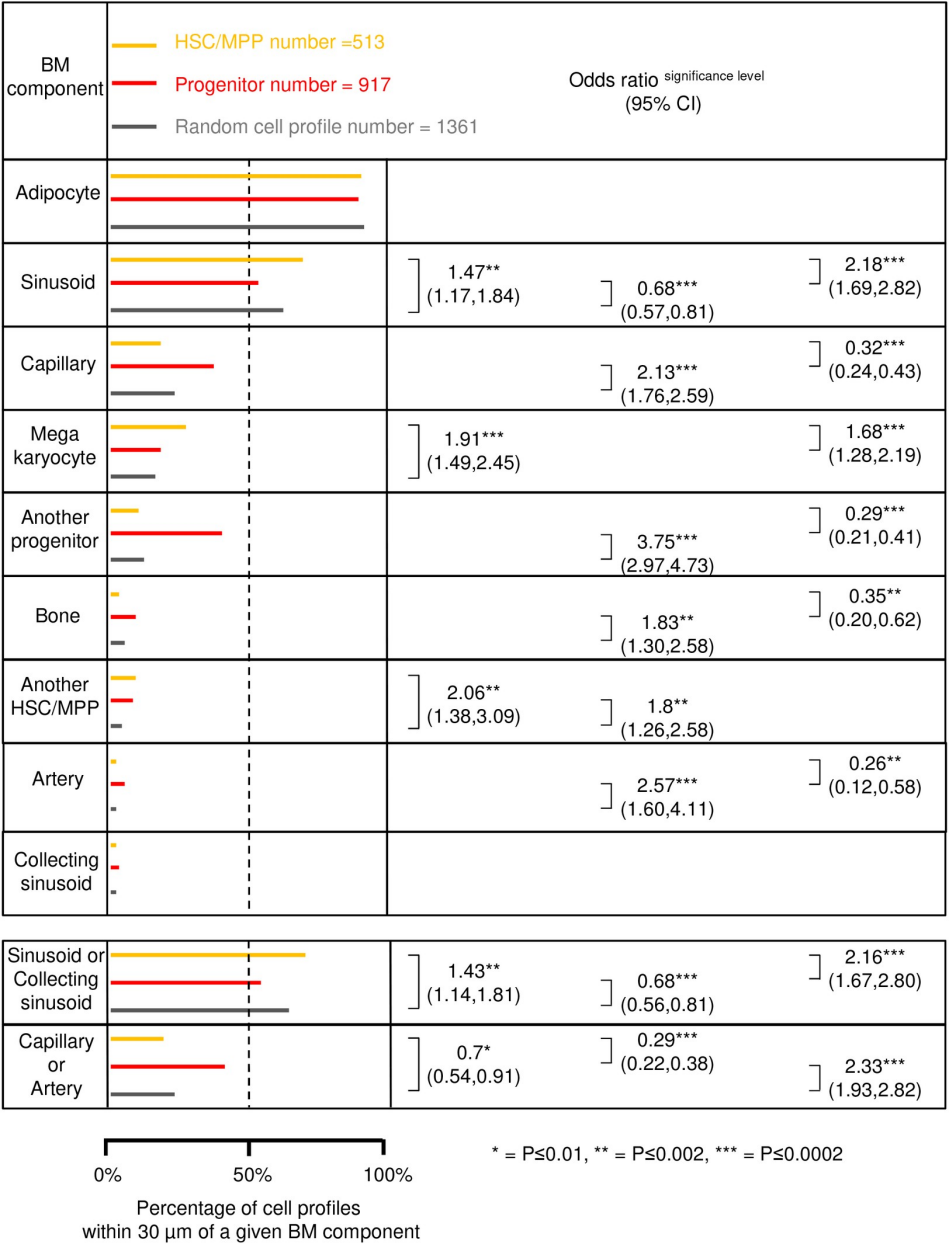
Relative likelihood of the HSCs/MPPs, the progenitors, and the random cell profiles to be in the presence of the indicated BM components. The length of the horizontal lines in yellow, red and grey shows the respective percentages of the HSCs/MPPs, the progenitors and the random cell profiles that show the indicated BM components within a distance of 30 μm. The nine BM components are ranked according to the level of the random cell profile presence within a distance of 30 μm, starting with the highest level of presence (adipocyte) and ending with the lowest level of presence (collecting sinusoids). Furthermore, the data for combination of sinusoids with collecting sinusoids, and of capillaries with arteries are also shown (at the lower part of the figure). The clustered logistic regression was used to compare the presence of each BM component (and the mentioned combinations) within a distance of 30 μm from each of the three cell profile categories. These comparisons were performed in pairs: The HSCs/MPPs vs. the random cell profiles, the HSCs/MPPs vs. the progenitors and the progenitors vs. the random cell profiles. The biopsy number was 10, and the cell profile number was 2791 (513 + 917 + 1361), when comparing the HSCs/MPPs and the progenitors to the random cell profiles, and 1430 (513 + 917) when comparing the HSCs/MPPs to the progenitors. When significant, the OR, the confidence interval, and the level of significance are indicated. * P ≤ 0.01, ** P ≤ 0.002, *** P ≤ 0.0002.

**Fig 4:**
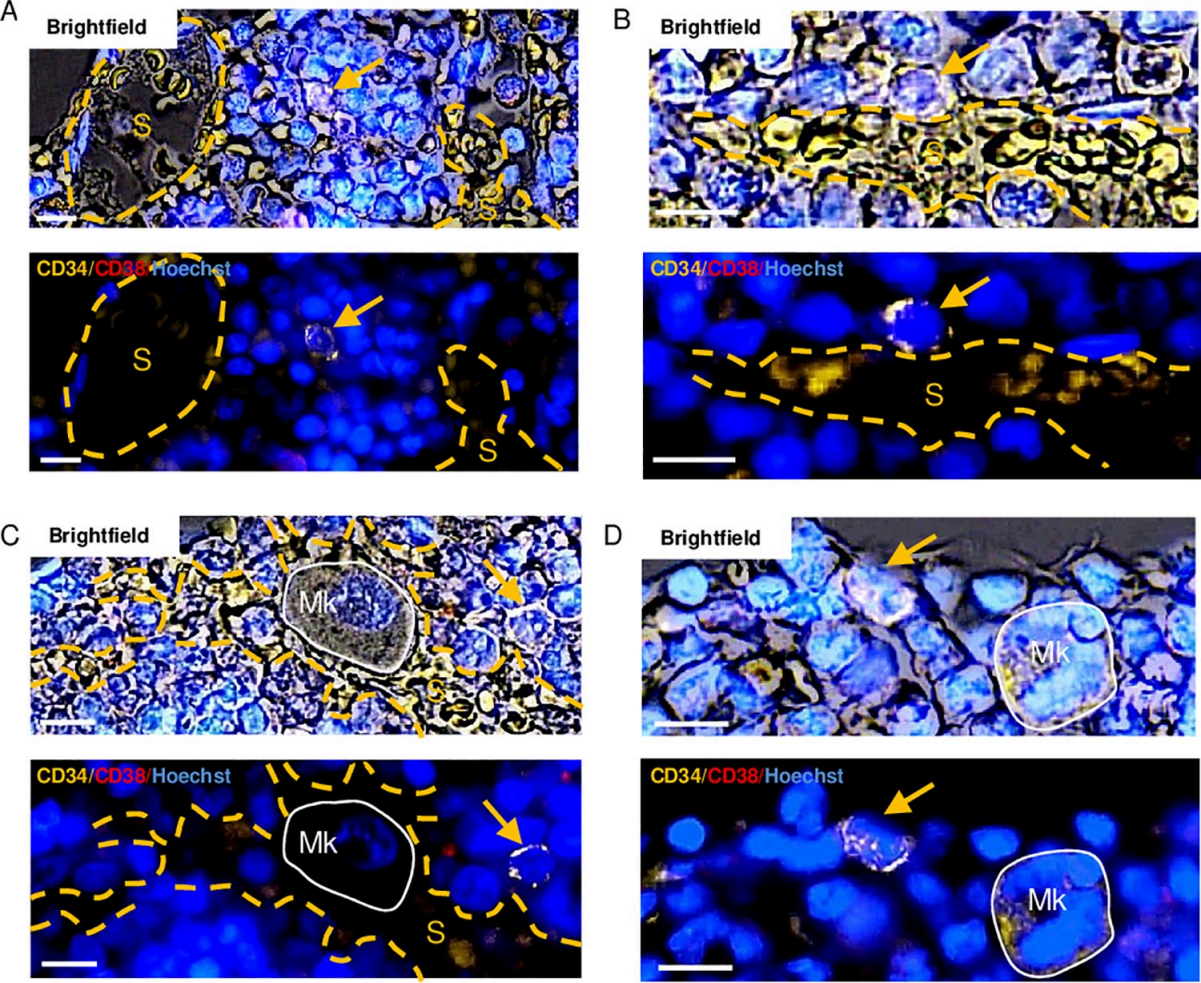
Illustrations of HSCs/MPPs that are positioned within a distance of 30 μm from sinusoids and/or megakaryocytes. The representative images are obtained from four different biopsies that were multiplex-stained as indicated. They are shown in brightfield (upper images) and pure fluorescence (lower images). (A) Presence of two different sinusoids (S and dashed yellow lines) within a distance of 30 μm from an HSC/MPP (yellow arrow). (B) Contact between a sinusoid and an HSC/MPP. (C) Presence of both a sinusoid and a megakaryocyte (Mk and white solid line) within a distance of 30 μm from an HSC/MPP. (D) Presence of a megakaryocyte within a distance of 30 μm from an HSC/MPP. Scale bars = 10 μm.

**Fig 5:**
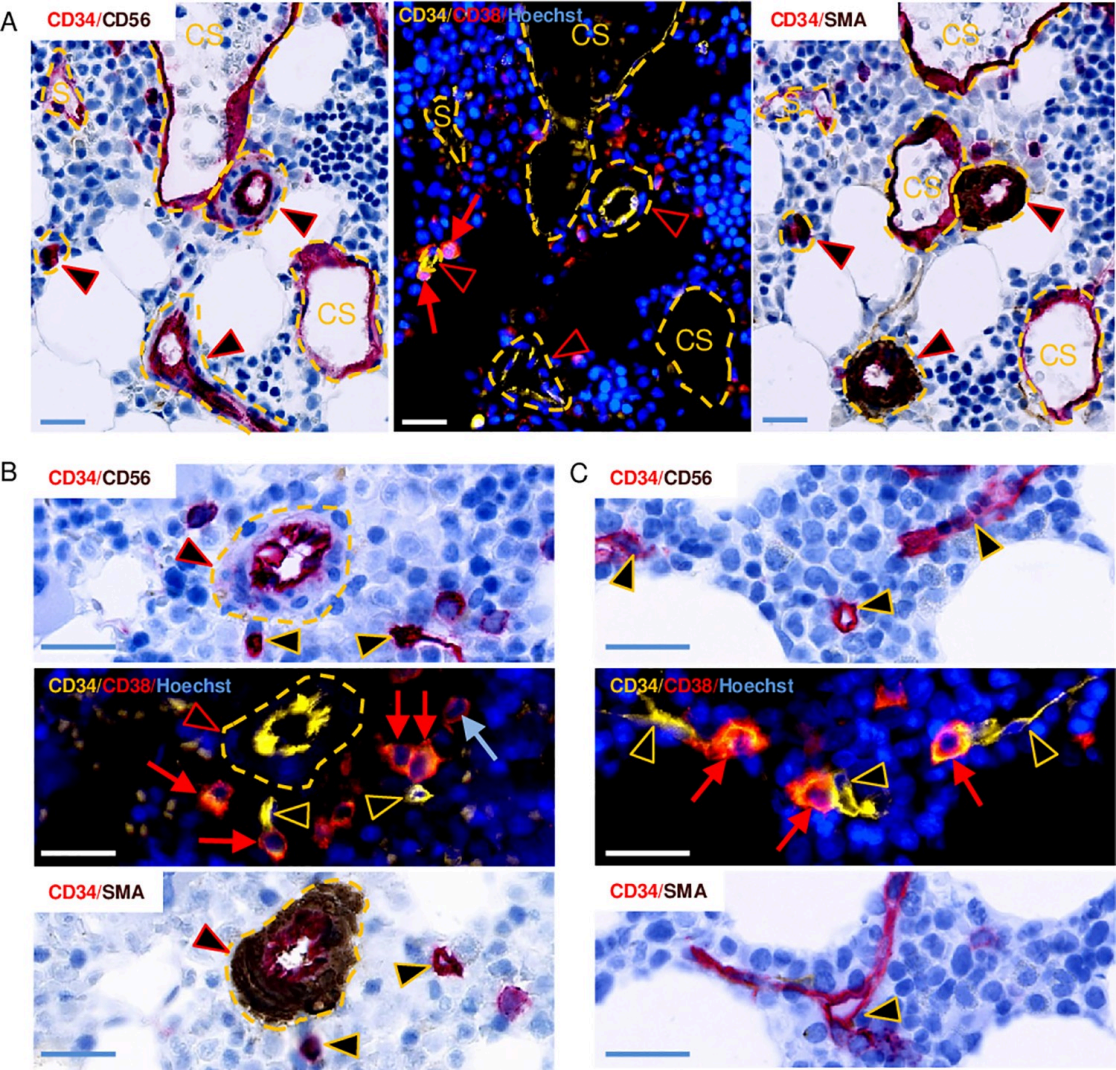
Illustrations of vascular structures including arteries and capillaries that are positioned within a distance of 30 μm from progenitors. The representative images are obtained from three different biopsies that were multiplex-stained as indicated. The three panels (A, B, C) each show three adjacent sections. Each pure fluorescence staining is shown between two immunohistochemistry stainings. (A) Two progenitors (red arrows at the left of the middle image) in contact with a small artery, three CD34^+^/CD38^-^/SMA^+^ and CD56^-^ arteries (red arrowheads), a patchy CD34-stained sinusoid (S and dashed yellow line), and two (left and middle), and three (right) CD34-stained collecting sinusoids (CS and dashed yellow line). (B) Four progenitors and a more differentiated cell profile (blue arrow), a CD34^+^/CD38^-^/SMA^+^ and CD56^-^ artery, and two capillary cross-sections (yellow arrowheads). (C) Contact between three progenitors and several segments of a capillary that ramifies in different directions. Note that the capillary has a narrow lumen and that is does not have a continuous layer of SMA^+^ SMCs. Scale bars = 10 μm.

The sinusoids are very present in the BM as they are very commonly found in the vicinity of the random cell profiles (Fig 3). The sinusoids are thin-walled vessels with CD34-patchy stained endothelial cell profiles, and they are shaped by the surrounding BM and have an erythrocyte-containing lumen (Figs 1 and 4). Interestingly, compared to both the random cell profiles and to the progenitors, the HSCs/MPPs were significantly more likely to have a sinusoid within a distance of 30 μm (ORs 1.47 and 2.18, respectively). Moreover, compared to the random cell profiles, the progenitors were significantly less likely to have a sinusoid in their BM microenvironment (OR 0.68) (Fig 3). The megakaryocytes have a distinct morphology and were relatively often present within 30 μm of the random cell profiles (Figs 3 and 4). Compared to both the random cell profiles and to the progenitors, the HSCs/MPPs were significantly more likely to have a megakaryocyte within a distance of 30 μm (ORs 1.91 and 1.68, respectively). (Fig 3). Here, the HSCs/MPPs and the progenitors include cell profiles at different maturation stages. So, the HSCs/MPPs and the progenitors were, compared to the random cell profiles, significantly more likely to have another HSC/MPP within a distance of 30 μm (OR 2.06 and 1.8, respectively) (Fig 3). The adipocytes were the BM component with the highest presence, while the collecting sinusoids were the least present (Fig 3). Compared to the sinusoids, the collecting sinusoids were larger, more ballooned, stained more intensively with CD34, and had frequent SMA^+^ pericytes (Fig 5). The HSCs/MPPs have the same chances as do the progenitors and the random cell profiles to have an adipocyte as well as a collecting sinusoid in their microenvironment (Fig 3). Sinusoids and collecting sinusoids carry blood with a relatively low oxygen tension [24], and the flow is slow in these large diameter vessels [39]. Together they comprise the “venous side” of the BM vasculature. We show a “venous side” to “arterial side”, ratio of 2.5:1 (Fig 3). Compared to both the random cell profiles and to the progenitors, the HSCs/MPPs were significantly more likely to have a “venous side” vessel within a distance of 30 μm (ORs 1.43 and 2.16, respectively). In contrast, compared to the random cell profiles, the progenitors were significantly less likely to have a “venous side” vessel within a distance of 30 μm (OR 0.68) (Fig 3).

### The progenitors have greater chances than the HSCs/MPPs to have capillaries, other progenitors, bone surfaces or arteries in their microenvironment

Capillaries are small-sized vessels with a narrow lumen, an innermost layer of CD34-intensively-stained endothelial cell profiles, and an incomplete outer layer of pericytes (Fig 5).

**Fig 6:**
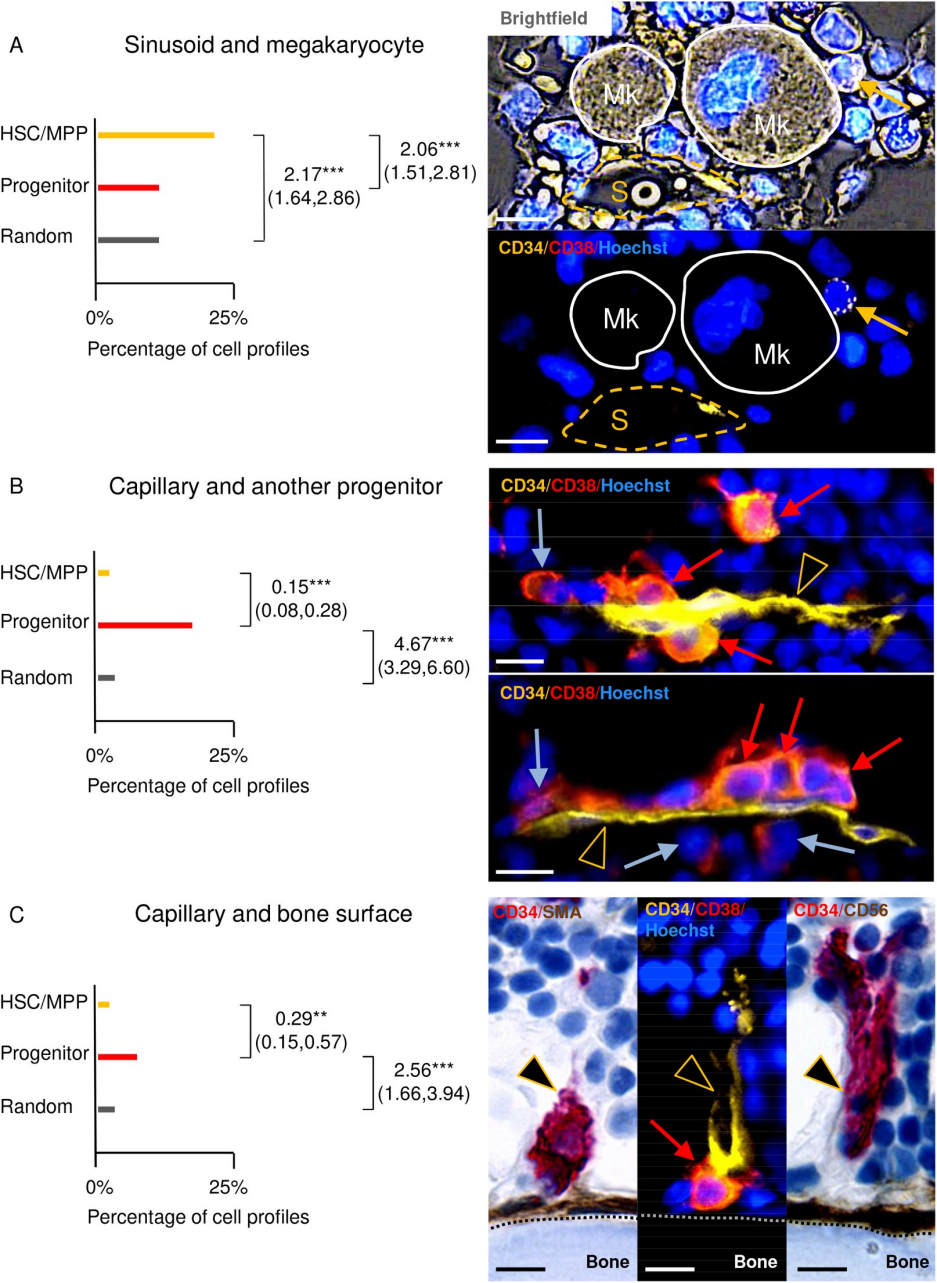
Relative likelihood of the HSCs/MPPs, the progenitors, and the random cell profiles to be in the presence of the indicated multicomponent BM components–and corresponding illustrations. (A, B, C graphs) The horizontal lines show the percentage of situations where a sinusoid and a megakaryocyte (A), a capillary and a progenitor (B), or a capillary and a bone surface (C) are respectively present within a distance of 30 μm from HSCs/MPPs (yellow), progenitors (red), and random cell profiles (grey). The clustered logistic regression was used to compare the presence of the three multicomponent microenvironments within a distance of 30 μm from each of the three cell categories. These comparisons were done in pairs: The HSCs/MPPs vs. the random cell profiles, the progenitors vs. the random cell profiles, and the HSCs/MPPs vs. the progenitors. The biopsy number was 10, and the cell profile number was 2791 (513 + 917 + 1361) when comparing the HSCs/MPPs and the progenitors to the random cell profiles, and 1430 (513 + 917) when comparing the HSCs/MPPs to the progenitors. When significant, the OR, the confidence interval, and the level of significance are indicated. ** P ≤ 0.002, *** P ≤ 0.0002. (A, B, C images) The representative images are from three different biopsies that were multiplex-stained, as indicated. They are shown in brightfield (upper image in A), pure fluorescence (lower image in A, the two images of B, and middle image in C), and as immunohistochemically-stained adjacent sections (left and right image in C). (A) An HSC/MPP (yellow arrow) in contact with a megakaryocyte (Mk and white solid line) and within a distance of 30 μm from another megakaryocyte and a sinusoid (S and dashed yellow line). (B) A capillary (yellow arrowhead) is positioned within a distance of 30 μm from three progenitors (red arrows) in both representative images, and the progenitors are positioned either in contact or within a distance of 30 μm from each other. Present are also respectively one (upper image) and two (lower image) more differentiated cells (blue arrows). (C) A progenitor in contact with both a capillary and a bone surface (dotted line). The bone surface is covered with SMA^+^ (left) and CD56^+^ (right) osteoblast lineage cells. Scale bars = 10 μm.

The capillaries are relatively common in the BM as they are commonly found in the microenvironment of the random cell profiles (Fig 3). Compared to the random cell profiles, the progenitors were significantly more likely to have a capillary within a distance of 30 μm (OR 2.13). In contrast, compared to the progenitors, the HSCs/MPPs were significantly less likely to have a capillary in their microenvironment (OR 0.32) (Fig 3). The arteries have an inner layer of CD34-intensively-stained, plump, endothelial cell profiles, and one or more layers of SMA^+^ SMCs (Fig 5). In the present study, the arteries were less often present than the capillaries (Fig 3). To be noted we found a capillary to artery ratio of 10:1 (Fig 3). Compared to the random cell profiles, the progenitors were significantly more likely to have an artery within a distance of 30 μm (OR 2.57). In contrast, the HSCs/MPPs were, compared to the progenitors, significantly less likely to have an artery in their microenvironment (OR 0.26) (Fig 3). Arteries and capillaries carry blood with a relatively high oxygen tension [24], and a high flow [39], they comprise the “arterial side” of the BM vasculature. Compared to the random cell profiles, the progenitors were significantly more likely to have an “arterial side” vessel within a distance of 30 μm (OR 2.33), whereas the HSCs/MPPs were significantly less likely (OR 0.7). Compared to the progenitors, the HSCs/MPPs were also significantly less likely to have an “arterial side” vessel in their BM microenvironment (OR 0.29) (Fig 3). Interestingly, the progenitors themselves represent a relatively common BM component (Fig 3), and compared to the random cell profiles, the progenitors were significantly more likely to have at least one other progenitor within a distance of 30 μm (OR 3.75), whereas the HSCs/MPPs compared to the progenitors were significantly less likely to have another progenitor in their BM microenvironment (OR 0.29) (Fig 3). At times we found up to eight other progenitors in the microenvironment of another progenitor. The bone surfaces are visualized both in the brightfield images (Fig 1) and in the adjacent immunohistochemically-stained sections (Fig 6); they represent a relatively uncommon BM component (Fig 3). Compared to the random cell profiles, the progenitors were significantly more likely to have a bone surface within a distance of 30 μm (OR 1.83). On the contrary, the HSCs/MPPs were, compared to the progenitors, significantly less likely to have a bone surface in their BM microenvironment (OR 0.35) (Fig 3).

### The HSCs/MPPs and the progenitors are associated with distinct combinations of BM components

The above analysis points to the fact that the HSCs/MPPs and the progenitors are each associated with distinct BM components. However, the term niche refers to a multicomponent BM microenvironment. Therefore, it is interesting that we show, that the compared to both the random cells profiles and the progenitors, the HSCs/MPPs were significantly more likely to have both a sinusoid and a megakaryocyte in their BM microenvironment (ORs 2.17 and 2.06, respectively) (Fig 6A left). Moreover, compared to the random cell profiles, the progenitors were significantly more likely to have both a capillary and another progenitor, or both a capillary and a bone surface, in their BM microenvironment (ORs 4.67 and 2.56, respectively) (Fig 6B left). In contrast, the HSCs/MPPs were, compared to the progenitors, significantly less likely to have these multicomponent BM microenvironments in their vicinity (ORs 0.15 and 0.29, respectively).The investigated multicomponent BM microenvironments next to the HSCs/MPPs and to the progenitors are illustrated in Figs 4–6.

## Discussion

Our knowledge regarding human HSPCs mainly comes from studies performed on aspirated cells. However, while aspirated cells are well suited for identification of cellular markers, no information regarding the multicomponent microenvironment next to HSPCs can be gained by this approach.

Here, we demonstrate that HSPCs can be identified on immunofluorescence-stained sections from human decalcified BM biopsies, and that the combination of information from immunofluorescence- and immunohistochemically-stained adjacent sections can be used to quantify their microenvironment. We show that the HSCs/MPPs have sinusoids and/or megakaryocytes in their microenvironment, while the progenitors have capillaries, other progenitors, bone surfaces and/or arteries. Interestingly, animal models lead to similar data.

In mice, Acar et al. [2] showed significantly fewer HSCs close to capillaries (transition zone vessels), arterioles and bone surfaces and significantly more HSCs close to sinusoids, compared to a population of random cell profiles. The sinusoids are needed for normal hematopoiesis, which is substantiated by several findings. In humans, there is an age dependent progressive conversion from hematopoietic BM to BM adipose tissue [40], and an age dependent loss of sinusoids [41]. The localization of HSCs next to sinusoids makes sense, since the sinusoids and the collecting sinusoids comprise the most hypoxic venous part of the BM [24], and brief exposure to oxygen induces human and murine HSCs to differentiate [23], while a mouse model showed preservation of HSC function by long-term hypoxia [42]. Worthy of note, we find a venous to arterial ratio of 2.5, which is in line with Brånemark’s findings [21] made more than 50 years ago. Moreover, we find a high capillary to artery ratio showing that the capillaries are the most common arterial vessel type in the human BM. The small caliber capillaries are designed for oxygenation, which fits with the finding of the highest oxygen tension next to the vessels with smallest vascular diameter in live animals [24]. Here, we show that the capillaries represent an important microenvironment for the progenitors, sometimes together with other progenitors or bone surfaces. Previously, we have shown that human bone surfaces are irrigated by a network of capillaries [25–27], while sinusoids are rarely found within a distance of 100 μm from the bone surface [26].

To our knowledge, this is the first study showing that the BM microenvironment of human HSCs/MPPs is enriched with megakaryocytes. Several murine studies have demonstrated a physiological and a functional relationship between HSCs and megakaryocytes [4,17,43,44]. Interestingly, Sanjuan-Pla et al. [44] showed that the megakaryocyte marker von Willebrand factor (vWF), can be used to identify platelet-biased murine HSCs. In addition to this, the trombopoietin receptor—the myeloproliferative leukemia (c-mpl)—is expressed both in human megakaryocytes and in human HSCs [43], and the c-mpl agonist, eltrombopag, expands the human HSPC population [45] and restores trilineage hematopoiesis in some patients with aplastic anemia [46]. Besides, sinusoids and megakaryocytes, the microenvironment of HSCs/MPPs is enriched with other HSCs/MPPs. Moreover, we report the highest ORs regarding enrichment of progenitors next to another progenitor and next to a capillary and another progenitor. The fact that the HSPCs tend to make up their own microenvironment, gives associations to murine findings, regarding clustering [14,47]. Moreover, it could be very interesting to investigate clustering in BM biopsies from patients with myeloid BM disease.

Together with morphological assessment we used CD34 and CD38 immunofluorescence-intensity and staining pattern to classify HSCs/MPPs and progenitors. A further sub-classification would demand inclusion of additional markers. One such marker could be CD90 [48], but during our preliminary investigations we were unable to find an antibody resulting in the necessary crisp, uniform, reproducible staining that we need. CD49f is another example of a marker that could be used to separate the HSCs from the MPPs [49], and it could be interesting to include CD49f in a future study. Moreover, CD45RA can probably be used to identify a population of CD34^+^/CD38^-^ early lymphoid progenitors [49]. Furthermore, it would be of great interest to extend the present characterization to other BM components, including macrophages [50], nerves [51], and MSCs [8,15,52]. A close physical association has been observed between sinusoids and MSCs, both in humans and in mice [8,15,52]. Murine studies have shown that normal MSCs are the major source of the chemokine CXCL12 [15]. Moreover, in a murine model of chronic myeloid leukemia deletion of CXCL12 in MSCs was shown to support the expansion of leukemic stem cells and suppress normal hematopoiesis, while deletion of CXCL12in endothelial cells led to a diminished expansion of leukemic stem cells [53]. The inclusion of CD271 [54,55] in a multiplex immunofluorescence-staining together with CD34 and CD38 in human BM biopsies both from normal individuals and patients with myeloid BM diseases could bring valuable insight into the relation between HSPCs, endothelial cells and MSCs in the human context.

The strengths of this study are: i) that we included healthy volunteers who are genetically heterogeneous, and represent a broad age span; ii) that the biopsies were obtained and processed according to standard procedures; iii) that the selected antibodies are commercially available; iv) that we identified the HSPCs using a combination of recognized markers and a set of morphological criteria on high quality material through a consensus agreement between health care professionals complementing each other’s different professional backgrounds.

Because of the evidence regarding the influence of aging on the HSPC microenvironment [14,56], we investigated whether it was possible to find any correlations between aging and HSPC localization; which we were not able to find. However, such a finding must be interpreted with great caution because we are dealing with a small sample size (biopsy wise), and at the same time with an uneven distribution of the different age groups.

In our study we found no significant association between HSPCs and the BM microenvironments that had the highest and lowest level of presence, e.g. the adipocytes, and the collecting sinusoids. What we found were highly significant associations for BM components with different levels of presence, e.g. the sinusoids and the arteries. Altogether, our data give no support for the findings that it should be the abundancy of the microenvironment in itself that dictates whether it associates to HSPC presence [57].

One might note that the ratio of HSCs/MPPs to progenitors differs in this study where we use a histological approach compared to flow cytometry. In order to understand the differences one should consider the different aspects of these approaches, including stereology and sampling. Firstly, the purpose of this study has been to investigate the association between HSPCs and BM components, and since we have not used a dissector, this study does not have the stereological design to make a quantification of cell numbers [58]. Thus, the reported cell numbers are estimates. Secondly, an unknown proportion of the HSCs/MPPs are sticking to the BM components, e.g. the sinusoids, implying that they will have a higher level of presence in BM sections compared to BM aspirates [59]. Thirdly, a small proportion of lymphoid progenitors are CD34^+^/CD38^-^/CD45RA^+^ [49], leading to a slight underestimation of the total number of progenitors. Fourthly, we have applied a set of morphological criteria which is not part of the flow cytometry set up. Fifthly, there is a variability in the intensity of staining amongst BM biopsies, which is one of the reasons why it is so important to use a statistical approach that takes both pooled data and data clustering into account.

In conclusion, we use a multiplex immunofluorescence-approach to detect human HSPC markers, and brightfield imaging as well as a multiplex immunohistochemistry-approach on adjacent sections to obtain knowledge about the positioning of these HSPCs. We show that the microenvironment of the HSCs/MPPs is significantly enriched in sinusoids and megakaryocytes, while the microenvironment of the progenitors is significantly enriched in capillaries, other progenitors, bone surfaces and arteries. Hereby, we expand several observations already made in mice to a human context. Our findings show some degree of conservation among species: (i) compared to random cells, HSCs are more likely to have a sinusoid in their microenvironment and less likely to have a bone surface or a capillary [2]; (ii) the HSC microenvironment is enriched in megakaryocytes [4,17]; (iii) there is a rich capillary network close to the bone surface [18]–fitting previous findings of a higher oxygen tension in the BM next to bone surfaces [24]. Our multicomponent analysis brings new insights into the organization of the human BM microenvironment. It could be very interesting to extend the analysis to include both additional BM components, such as macrophages, nerves, and MSCs in the physiological setting, and to expand our knowledge regarding the BM microenvironment in BM malignancies.

## Supporting information

S1 File (XLSX)
